# Improving influenza virological surveillance in Europe: strain-based reporting of antigenic and genetic characterisation data, 11 European countries, influenza season 2013/14

**DOI:** 10.2807/1560-7917.ES.2016.21.41.30370

**Published:** 2016-10-13

**Authors:** Eeva Broberg, Olav Hungnes, Brunhilde Schweiger, Katarina Prosenc, Rod Daniels, Raquel Guiomar, Niina Ikonen, Athanasios Kossyvakis, Francisco Pozo, Simona Puzelli, Isabelle Thomas, Allison Waters, Åsa Wiman, Adam Meijer

**Affiliations:** 1 European Centre for Disease Prevention and Control (ECDC), Stockholm, Sweden; 2 Norwegian Institute of Public Health, Oslo, Norway; 3 National Influenza Reference Centre, Robert Koch-Institute, Berlin, Germany; 4 Laboratory for Public Health Virology, National Laboratory for Health, Environment and Food Slovenia, Ljubljana, Slovenia; 5 Crick Worldwide Influenza Centre, The Francis Crick Institute, Mill Hill Laboratory, London, United Kingdom; 6 National Influenza Reference Laboratory, National Institute of Health Dr Ricardo Jorge, Lisbon, Portugal; 7 Viral Infections Unit, National Institute for Health and Welfare (THL), Helsinki, Finland; 8 National Influenza Reference Laboratory of Southern Greece, Hellenic Pasteur Institute, Athens, Greece; 9 Respiratory Viruses and Influenza Unit, National Center of Microbiology, Instituto de Salud Carlos III, Madrid, Spain; 10 National Influenza Centre, Instituto Superiore di Sanità, Rome, Italy; 11 National Influenza Reference Centre, Scientific Institute of Public Health, Brussels, Belgium; 12 National Virus Reference Laboratory, University College Dublin, Dublin, Ireland; 13 Public Health Agency of Sweden, Solna, Sweden; 14 National Institute of Public Health and the Environment, Bilthoven, the Netherlands

**Keywords:** influenza, influenza season, age, genetic clade, hospitalisation

## Abstract

Influenza antigenic and genetic characterisation data are crucial for influenza vaccine composition decision making. Previously, aggregate data were reported to the European Centre for Disease Prevention and Control by European Union/European Economic Area (EU/EEA) countries. A system for collecting case-specific influenza antigenic and genetic characterisation data was established for the 2013/14 influenza season. In a pilot study, 11 EU/EEA countries reported through the new mechanism. We demonstrated feasibility of reporting strain-based antigenic and genetic data and ca 10% of influenza virus-positive specimens were selected for further characterisation. Proportions of characterised virus (sub)types were similar to influenza virus circulation levels. The main genetic clades were represented by A/StPetersburg/27/2011(H1N1)pdm09 and A/Texas/50/2012(H3N2). A(H1N1)pdm09 viruses were more prevalent in age groups (by years) < 1 (65%; p = 0.0111), 20–39 (50%; p = 0.0046) and 40–64 (55%; p = 0.00001) while A(H3N2) viruses were most prevalent in those ≥ 65 years (62%*; p = 0.0012). Hospitalised patients in the age groups 6–19 years (67%; p = 0.0494) and ≥ 65 years (52%; p = 0.0005) were more frequently infected by A/Texas/50/2012 A(H3N2)-like viruses compared with hospitalised cases in other age groups. Strain-based reporting enabled deeper understanding of influenza virus circulation among hospitalised patients and substantially improved the reporting of virus characterisation data. Therefore, strain-based reporting of readily available data is recommended to all reporting countries within the EU/EEA.

## Background

Influenza virological surveillance data, including characteristics of circulating viruses, are collected to describe the annual occurrence of influenza virus (sub)types and lineages for selection of vaccine components for the following season. Virological surveillance also supports epidemic and pandemic preparedness with detection of emerging influenza viruses. European Union and European Economic Area (EU/EEA) countries report influenza surveillance data on a weekly basis during influenza seasons as part of the World Health Organization (WHO) Global Influenza Surveillance and Response System (GISRS) [1] to describe the antigenic character and genetic makeup of circulating viruses [2]. Surveillance at the EU/EEA level is carried out by the European Influenza Surveillance Network (EISN) and data are collected on a weekly basis in The European Surveillance System (TESSy) under the coordination of the European Centre for Disease Prevention and Control (ECDC) [3,4].

Through the sentinel and non-sentinel surveillance systems in EU/EEA countries, subsets of viruses, detected across the season from different geographic locations and from different demographic groups, are further characterised by the National Influenza Centres (NICs) for their antigenic and genetic properties, and antiviral susceptibility. Smaller subsets of influenza virus positive specimens and virus isolates are sent by NICs to a WHO Collaborating Centre for Influenza Reference and Research (CC), mainly the WHO CC London, United Kingdom, for detailed characterisation.

Historically, EU/EEA countries have reported aggregate influenza virus detections by type and subtype, together with influenza-like illness (ILI)/acute respiratory infection (ARI) consultation rate data from sentinel primary healthcare providers to TESSy. Antigenic and genetic characteristics for a subset of these viruses, aggregated by week of sampling, have been reported according to predefined categories, based on reference viruses representing antigenic and genetic similarity to either vaccine viruses or known antigenic/genetic ‘drift’ variants. Due to the aggregate format, patient information (e.g. age, sex, vaccination or hospitalisation status) was not collected. The majority of countries reported age group-specific ILI/ARI rates without being able to link them to age-specific virological data. In 2004, strain-based reporting of influenza antiviral susceptibility with epidemiological, demographic and clinical information was introduced [5]. In the 2007/08 influenza season, this new system facilitated rapid assessment of the spread of former seasonal A(H1N1) influenza viruses showing clinical resistance to oseltamivir due to neuraminidase (NA) H275Y amino acid substitution [6].

Although there have been earlier studies on severity and its association with influenza subtypes [7-10], there is limited evidence of risk factors for severe influenza or influenza complications due to specific subtypes and viruses [11]. To assess the disease burden in different patient risk groups caused by influenza viruses of various (sub)types with particular antigenic and genetic characteristics, it is crucial from the public health perspective to have detailed information about the distribution of specific viruses in different risk groups. This study piloted the integrated collection of strain-based antigenic and genetic characterisation data and epidemiological, demographic and clinical information.

The objectives were: (i) to test the feasibility of collecting influenza virus strain-based antigenic and genetic data; and (ii) to assess the collected data and explore the benefits of non-aggregate strain-based reporting.

## Methods

### Data collection

Respiratory specimens were obtained in the participating countries as part of their routine influenza surveillance activities from week 40/2013 to week 39/2014. Sentinel general practitioners swabbed patients with ILI and/or another ARI, with most meeting the EU case definition for ILI and/or ARI [12], depending on the country’s choice of syndrome under surveillance and following the nationally agreed sampling protocol. Non-sentinel specimens, mainly from hospital laboratories, were also included. All specimens were analysed for the presence of influenza virus, by real-time RT-PCR, at the local laboratory or the NIC. If specimens were first analysed at a local laboratory, all or a subset of influenza-virus-positive specimens or virus isolates were sent to the NIC for further analysis of subtype or lineage, antigenic characterisation by haemagglutination inhibition assay, and genetic characterisation by sequencing of haemagglutinin (HA) genes. All participating laboratories take part in regular external quality assessments of rapid detection, virus culture, antigenic and genetic characterisation and antiviral susceptibility analysis [13]. Within EISN, a target of characterising ca 10% of influenza detections has been agreed, although depending on predominant virus (sub)type and intensity of the epidemic, it is valid to characterise less than 10%. In addition, NICs sent smaller subsets of specimens and virus isolates to the WHO CC in London for more detailed characterisation. When selecting specimens for characterisation, laboratories were expected to include specimens with sufficient viral load, based on their resources from all (sub)types, from different age groups, surveillance systems, geographical locations and phases of the epidemic [14].

As part of the existing reporting scheme, countries reported weekly aggregate virological influenza surveillance and antigenic and genetic characterisation data to ECDC. Prefixed, coded reporting categories defined by WHO CC London were used for antigenic and genetic characteristics which included vaccine viruses and additional non-vaccine reference viruses with specific antigenic properties or specified HA amino acid substitutions and phylogenetic clade (see Table 1 for the categories).

**Table 1:** Strain-based reporting: numbers of influenza viruses by antigenic group and genetic clade, 11 European countries, influenza season 2013/14

Antigenic group	Number (%)	Genetic clade	Number (%)	Number of viruses with both antigenic and genetic data
A/California/7/2009 (H1N1)pdm09	306 (46)	A/St Petersburg/27/2011 (H1N1)pdm09	513 (46)	72
A(H1N1)pdm09 not categorised	0 (0)	A(H1N1)pdm09 not categorised	0 (0)	0
A/Texas/50/2012 (H3N2)	305 (46)	A/Texas/50/2012 (H3N2)	519 (46)	52
A(H3N2) not categorised	11 (2)	A(H3N2) not categorised	0 (0)	4
B/Brisbane/60/2008 (Victoria-lineage)	11 (2)	B/Brisbane/60/2008 (Victoria-lineage)	13 (1)	4
B (Victoria-lineage) not categorised	0 (0)	B (Victoria-lineage) not categorised	0 (0)	0
B/Massachusetts/02/2012 (Yamagata-lineage)	23 (3)	B/Massachusetts/02/2012 (Yamagata-lineage)	22 (2)	11
NA	NA	B/Wisconsin/1/2010 (Yamagata-lineage)	50 (4)	0
B (Yamagata-lineage) not categorised	3 (0.5)	B (Yamagata-lineage) not categorised	0 (0)	0
**Total**	**659**	**Total**	**1,117**	**143**

NA: not applicable.

In addition, for this pilot study, all EU/EEA countries were invited to submit antigenic and/or genetic characterisation data in strain-based format. The virus name, e.g. A/Netherlands/2245/2013, acted as a unique identifier and duplicated data from national and WHO CC sources were merged. The epidemiological data included variables: age, complication diagnosis, date of onset, exposure to antiviral drugs, sex, hospitalisation, immunocompromised status, outcome, probable country of infection and vaccination status. All data for the 2013/14 influenza season were extracted from TESSy on 15 January 2015. In addition, HA-gene sequences of viruses for which database accession numbers were reported were retrieved from the Global Initiative on Sharing All Influenza Data (GISAID) EpiFlu database.

### Data analysis

#### Feasibility of reporting

Feasibility of strain-based reporting was assessed through the pilot, looking at country-wide distribution among participating countries and data completeness. We also received comments on the feasibility of the reporting by questionnaire.

#### Detection and characterisation data

Detection and characterisation data were plotted by week of specimen collection over the influenza season (week 40/2013 to week 39/2014) and timing of aggregate and strain-based antigenic and genetic characterisations were compared between detections from both sentinel and non-sentinel data sources.

#### Genetic data

Nucleic acid sequences encoding the HA1 subunit were subjected to cluster analysis of maximum-likelihood phylogenetic trees using BioNumerics 7.5 software. Furthermore, encoded HA1 subunit sequences were checked for match to the signature amino acid substitutions of the genetic categories that individual viruses had been ascribed to. The resulting phylogenetic trees were checked for misattributed viruses, as well as for apparent clade patterns beyond the resolution of the categories provided in the TESSy reporting scheme. The European Reference Laboratory Network for Human Influenza (ERLI-Net) reference HA1 encoding sequence sets provided by WHO CC for the 2013/14 season were used as reference sequences in the analysis. To better understand the ongoing evolution of the viruses and in order to check for the presence of groups that predominated in the following season, two A(H3N2) and one B/Yamagata-lineage ERLI-Net reference viruses defined for the subsequent 2014/15 season were also included: A/Switzerland/9715293/2013(H3N2) (group 3C.3a); A/Hong Kong/5738/2014(H3N2) (group 3C.2a); and B/Phuket/3373/2013 (clade 3).

#### Antiviral susceptibility data

Extended virus characterisation was achieved by including antiviral susceptibility data in the analysis. To standardise interpretation and reporting of influenza virus susceptibility to the neuraminidase (NA) inhibitors (NAIs) oseltamivir and zanamivir, WHO-Antiviral Working Group definitions, based on half maximal inhibitory concentration (IC_50_),were used [15]. Raw IC_50_ data were converted into relative fold-change values compared with the median of all data by virus type or subtype and NAI to facilitate pooled analysis of the data from all laboratories [16]. As influenza B virus IC_50_ data varied widely between laboratories, the fold-changes for influenza B viruses were calculated by reporting laboratory. IC_50_ fold-change data were generated to classify the viruses as with normal inhibition (NI), reduced inhibition (RI) or highly reduced inhibition (HRI). Amino acid substitution data were analysed against published data on specific amino acid substitution in the M2 and NA proteins previously associated with resistance to adamantane M2 ion channel blockers and RI or HRI by NAIs (oseltamivir and zanamivir), respectively [17].

#### Epidemiological data and statistical analysis

Patients were stratified into the following age groups: < 1 year, 1–5 years, 6–19 years, 20–39 years, 40–64 years and ≥ 65 years. Distribution of sex by age group was tested for significance using the nonparametric Kruskal-Wallis test. Distribution of genetic clades in different age groups was compared by Dunn’s test (multiple pairwise comparisons using rank sums) with Bonferroni adjustment. The level of significance was set at p < 0.05.

## Results

### Participating countries, data completeness and feasibility

Eleven of 30 EU/EEA countries participated in this pilot: Belgium, Finland, Germany, Greece, Ireland, Italy, the Netherlands, Norway, Portugal, Spain and Sweden. However, Belgium did not report patient age and sex, so Belgian cases were excluded from epidemiological analysis. Data completeness is shown in Table 2. All reporting laboratories found the reporting feasible and recommended the use of it to other laboratories in the questionnaire (data not shown).

**Table 2:** Data completeness for reported variables, strain-based reporting of antigenic and genetic characterisation data for influenza viruses, 11 European countries, influenza season 2013/14 (n=1,633)

Variable	Number (%)
Virus (sub)type	1,633 (100)
Sex	1,577 (97)
Age	1,547 (95)
Hospitalisation status	1,147 (70)
Date of onset	1,052 (64)
Vaccination status	798 (49)
Patient given or not given antivirals before collection of specimen	725 (44)
Probable country of infection	669 (41)
Immunocompromised status	521 (32)
Outcome (alive/dead)	521 (32)
Complication diagnosis	219 (13)
Household member given or not given antivirals before collection of specimen	75 (5)

#### Influenza virus detections vs characterisations

Participating countries detected 15,669 influenza viruses during the 2013/14 season of which 3,920 (25%) were from sentinel and 11,749 (75%) from non-sentinel sources (Table 3). The same countries submitted strain-based data for 1,633 influenza viruses (10% of the detections): 586 (36%) were from sentinel sources and 1,037 (64%) from non-sentinel sources (Table 3). For 10 viruses (1%), the source was not declared.

**Table 3:** Number of reported influenza viruses by (sub)type and source, 11 European countries, influenza season 2013/14

Influenza	Aggregate (virus detections)			Strain-based (virus characterisations)				Characterised viruses as a proportion of detections (%)		
	Sentinel	Non-sentinel	Total	Sentinel	Non-sentinel	Unknown	Total	Sentinel	Non-sentinel	Total
A(H1N1)pdm09	2,089	7,690	**9,779**	237	505	5	**747**	11.3	7	**8**
A(H3N2)	1,714	3,219	**4,933**	311	464	4	**779**	18.1	14	**16**
B(lineage not determined)	60	663	**723**	0	0	0	**0**	NA	NA	**NA**
B(Victoria)	7	9	**16**	9	11	0	**20**	129^a^	122^a^	**125^a^**
B(Yamagata)	50	168	**218**	29	57	1	**87**	58	34	**40**
Total detections (aggregate) or reports (strain-based)	3,920	11,749	**15,669**	586	1,037	10	**1,633**	15	9	**10.4**
Number of specimens tested for influenza	11,631	112,571	**124,202**	NA^b^						

NA: not applicable.

^a^ > 100% as some of the B(lineage not determined) viruses were characterised at later dates and then reported by influenza B virus lineage.

^b^ This category is not applicable to strain-based reporting as only influenza-positive specimens can be reported on; the number of ‘specimens’ is the total number of reports.

In both sentinel and non-sentinel specimens, influenza types A and B were detected and all type A viruses were subtyped. Participating countries detected 9,779 (62%) A(H1N1)pdm09, 4,933 (32%) A(H3N2) and 957 (6%) type B viruses. Of the B viruses, lineage was determined for 234 (24%), and of these, 218 (93%) were B/Yamagata-lineage (Table 3).

Of the 1,633 viruses reported in the strain-based system, 747 (46%) were A(H1N1)pdm09, 779 (48%) A(H3N2) and 107 (7%) type B viruses (Table 3). A slightly higher proportion of viruses were characterised from sentinel than from non-sentinel sources (Table 3).

#### Antigenic and genetic characterisation

Of the 1,633 viruses characterised, 516 (32%) were only characterised antigenically, 974 (60%) only genetically and 143 (9%) both antigenically and genetically (Table 1). For the latter, the antigenic and genetic characterisation data were consistent. The participating countries contributed unequally to the antigenic and genetic characterisation data. Germany submitted 300 (45%) and Portugal 151 (23%) of the 659 antigenic characterisation records, with other countries contributing between one (0.2%) and 58 (9%) of the records while Finland and Sweden submitted no antigenic data. Spain contributed the most genetic characterisation data, accounting for 513 (46%) of the 1,117 records, with other countries providing details on between 10 (1%) and 125 (11%) viruses. Italy provided no genetic characterisation data.

Antigenically and genetically characterised viruses fell mainly in the A/California/7/2009 (H1N1)pdm09-like (in the A/St Petersburg/27/2011 subgroup) and A/Texas/50/2012(H3N2)-like reporting categories (46% in each category), the A(H1N1) and A(H3N2) components of the 2013/14 northern hemisphere influenza vaccines. Eleven A(H3N2) viruses were reported as ‘not categorised’ antigenically and would therefore be low reactors or not reacting with antiserum against the reference virus. For four of these, the genetic category was assigned as A/Texas/50/2012(H3N2). For the remaining seven viruses, no additional genetic information was available.

Type B viruses were detected in smaller numbers than influenza A viruses, and only 37 type B viruses were characterised antigenically: 11 B/Victoria-lineage viruses as B/Brisbane/60/2008-like and 26 B/Yamagata-lineage viruses as B/Massachusetts/02/2012-like (n = 23; 2013/14 vaccine component) or as ‘not categorised’ (n = 3), respectively. Of the 85 B viruses characterised genetically, 13 were B/Victoria-lineage viruses, and of the 72 B/Yamagata-lineage viruses, 22 and 50 fell within clades represented by B/Massachusetts/02/2012 (clade 2) and B/Wisconsin/1/2010 (clade 3), respectively.

#### Progression of the influenza season and timing of characterisations

**Figure 1:**
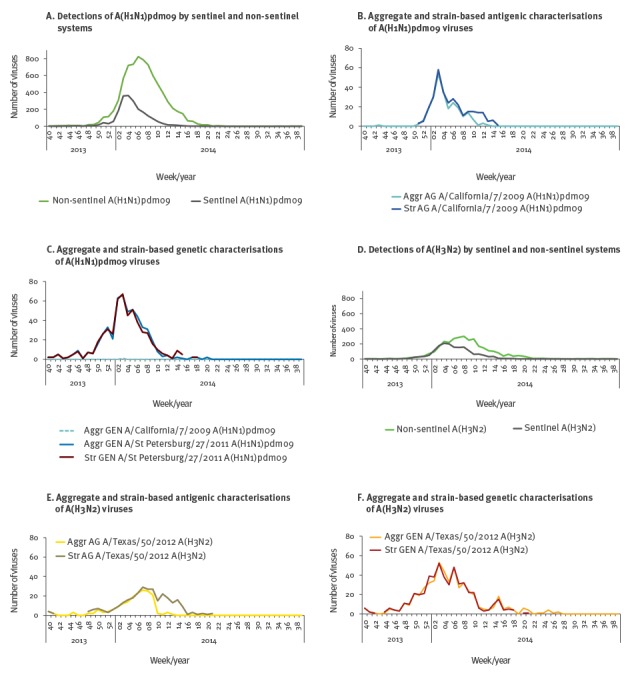
Detections and characterisations by influenza A virus subtype and surveillance system, by week of specimen collection, strain-based reporting of antigenic and genetic characterisation data, 11 European countries, influenza season 2013/14

AG: antigenic; aggr: aggregate; GEN: genetic; str: strain-based.

To analyse the distribution of characterisations over the influenza season, we compared the number of characterisations and detections by weeks. The influenza season in the 11 participating countries occurred from week 49/2013 to week 18/2014. The highest numbers of detections of A(H1N1)pdm09 viruses were reported in week 04/2014 for sentinel sources and week 06/2014 for non-sentinel sources. A(H3N2) virus detections peaked in week 04/2014 for sentinel and week 08/2014 for non-sentinel sources (Figure 1). Although B viruses were detected throughout the season, detections peaked in week 15/2014, originating mostly from non-sentinel sources (data not shown).

For A(H1N1)pdm09 viruses, similar reporting patterns were seen for both phenotypically and genetically characterised strains, with the majority being reported in weeks 01–11/2014. Similarly, for A(H3N2) viruses, the majority of strain-based reports were for viruses detected in weeks 02–12/2014. Although low, the highest numbers of influenza B detections occurred during weeks 04–21/2014. Antigenic characterisations of B viruses were reported for weeks 40/2013–20/2014 and genetic characterisations for weeks 40/2013–27/2014 (data not shown). Overall, the number of antigenic and genetic characterisations followed the season progression for all virus (sub)types.

#### Analysis of available sequence data

All 596 HA sequences (271 H1, 287 H3, 7 B/Victoria and 31 B/Yamagata), for which accession numbers had been provided in TESSy, were retrieved. Analysis of genetic group-defining amino acid substitutions and phylogenetic clades revealed that all sequences available for this analysis were categorised in accordance with the reporting scheme. However, a number of sequences (71 A(H1N1)pdm09, 54 A(H3N2) and 6 B/Yamagata) were excluded from the phylogenetic analysis because they did not cover either full-length coding regions of HA1 subunit for influenza A(H1) and (H3), or HA1 amino acids 28–314 for type B/Victoria or 22–339 for type B/Yamagata.

**Figure 2:**
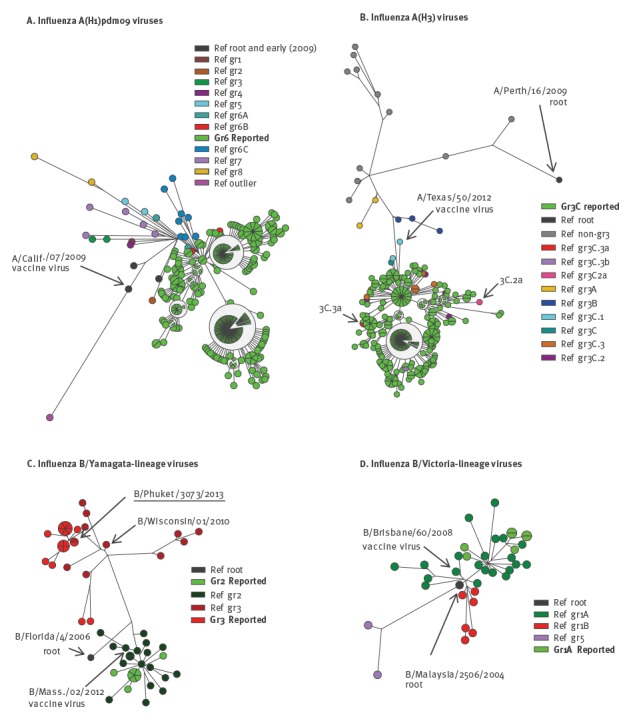
Phylogenetic and cluster analysis of available haemagglutinin 1 sequences, strain-based reporting of antigenic and genetic characterisation data for influenza viruses, 11 European countries, influenza season 2013/14 (n=596)

For A(H1N1)pdm09 viruses, all 271 sequences analysed were correctly attributed to the broad genetic group represented by A/St.Petersburg/27/2011, known as group 6 in global influenza surveillance terminology. No further distinction was available in the reporting scheme. However, all viruses belonged to subgroup 6B, represented by reference viruses such as A/South Africa/3626/2013 and A/Norway/2417/2013 (Figure 2A).

ERLI-Net: European Reference Laboratory Network for Human Influenza; HA: haemagglutinin; Ref: ERLI-Net reference sequences.

Maximum-likelihood analysis of HA1 subunit sequences is shown. The reported haemagglutinin sequences and ERLI-Net reference sequences were colour-coded according to their attributed genetic categories. Identical sequences are displayed as segmented node circles where the circle area and number of segments represent the number of viruses.

All A(H3N2) viruses were reported as belonging to the group represented by A/Texas/50/2012 (the 2013/14 vaccine virus), a subgroup 3C virus subsequently defined as representing the 3C.1 subdivision after the 2013/14 influenza season. Amino-acid signature and phylogenetic cluster analysis confirmed that all available sequences were correctly attributed to subgroup 3C, but distributed within two subdivisions, 3C.2 and 3C.3. One virus (A/Norway/466/2014) clustered with the antigenic drift variant A/Switzerland/9715293/2013 which was representative of genetic subgroup 3C.3a viruses and is the recommended A(H3N2) vaccine virus for the 2015/16 influenza season (Figure 2B). No sequences clustered with another genetic subgroup, 3C.2a, associated with antigenic drift in the course of the subsequent 2014/15 influenza season.

B/Yamagata-lineage viruses fell within the two circulating clades represented by B/Massachusetts/02/2012 (clade 2; vaccine virus 2013/14) and B/Wisconsin/01/2010 (clade 3). The majority was attributed to clade 3. Consistent with this, available sequences clustered with these two groups and were in all instances correctly attributed (Figure 2C). Notably, the majority of clade 3 sequences closely matched a recent reference virus, B/Phuket/3073/2013, recommended for use in southern hemisphere 2015 and northern hemisphere 2015/16 influenza vaccines. All seven B/Victoria-lineage sequences clustered with the clade 1A reference sequences of which the vaccine virus, B/Brisbane/60/2008, is representative (Figure 2D)

#### Antiviral susceptibility

Of the 1,633 viruses with antigenic and/or genetic characterisation data, 678 (42%) were tested for neuraminidase inhibitor (NAI) susceptibility using genetic and/or phenotypic methods: 349 A(H1N1)pdm09, 264 A(H3N2), 54 B/Yamagata-lineage and 11 B/Victoria-lineage viruses. One A(H1N1)pdm09 virus carrying neuraminidase (NA) I223R amino acid substitution showed reduced inhibition (RI) by oseltamivir. Two others showed RI by zanamivir, only one of which was sequenced and shown to carry NA S247I substitution. One virus carried NA H275Y substitution which has been associated with highly reduced inhibition (HRI) by oseltamivir but it was not tested phenotypically. One A(H3N2) virus showed RI by oseltamivir and zanamivir and one by zanamivir only. Both viruses were sequenced but no amino acid substitutions previously or potentially associated with RI were identified. One B virus showed RI by zanamivir (sevenfold) but no amino acid substitution previously or potentially associated with RI was identified. For 80 cases with antiviral susceptibility data, antiviral treatment with oseltamivir up to 14 days before specimen collection was reported, including one case infected with an A(H3N2) virus showing RI by zanamivir. All other cases with indications of being infected with viruses showing RI or HRI by a NAI, for which antiviral exposure status was reported, had not received antivirals before specimen collection. One case infected with A(H1N1)pdm09 carrying NA S247N substitution was exposed to oseltamivir through a treated household contact.

#### Demographic and patient data

##### Sex and age

**Figure 3:**
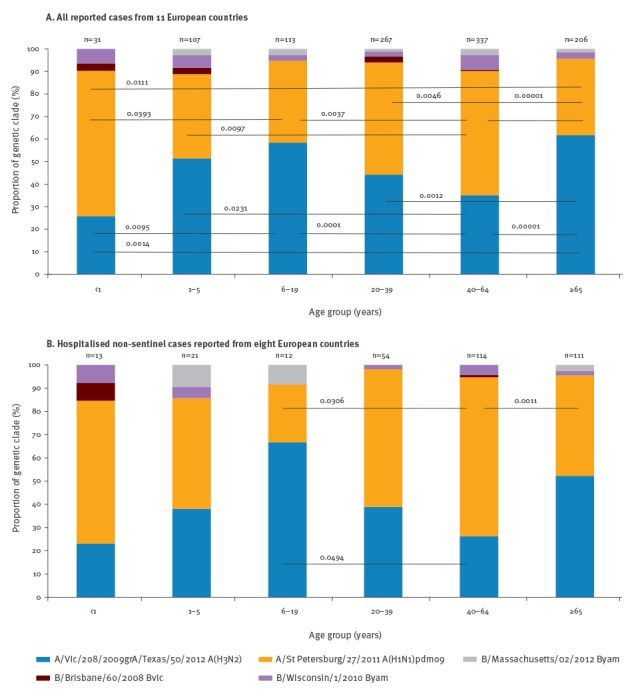
Proportions of influenza virus genetic clades by patient age, strain-based reporting of antigenic and genetic characterisation data from (A) all reported cases with age from 11 European countries (n = 1,061) and (B) hospitalised non-sentinel cases reported from eight European countries (n = 325), influenza season 2013/14

The majority of the 1,547 cases for which age was reported by 11 countries were adults aged 20–64 years (53%). The sex distribution did not vary significantly across age groups (50% female and male, n = 1,535; p = 0.1611). Age and genetic clade was available for 1,061 cases. A/St Petersburg/27/2011-like A(H1N)pdm09 viruses affected age groups < 1 year, 20–39 years and 40–64 years (65%, p = 0.0111; 50%, p = 0.0046; 55%, p = 0.00001, respectively) more than the  ≥ 65 years age group (34%). A/Texas/50/2012-like A(H3N2) viruses affected more of the  ≥ 65 year olds (62%; p = 0.0012) than 20–39 year olds (44%). A/Texas/50/2012-like A(H3N2) viruses affected the age groups < 1 year (26%; p = 0.0014) and 40–64 years (35%; p = 0.00001) less than the age group ≥ 65 years (Figure 3A).

Distribution of genetic clades between different age groups compared by Dunn’s test with Bonferroni adjustment, p values indicated for each statistically significant comparison.

The 11 countries in panel A are Belgium, Finland, Germany, Greece, Ireland, Italy, the Netherlands, Norway, Portugal, Spain and Sweden.

The eight countries in panel B are Finland, Greece, Ireland, the Netherlands, Norway, Portugal, Spain and Sweden.

#### Hospitalisation

Hospitalisation status and influenza virus subtype were reported for 1,147 (70%) of 1,633 cases. Of these, 672 cases were reported from non-sentinel sources and included reporting from 10 countries (Finland, Germany, Greece, Ireland, Italy, the Netherlands, Norway, Portugal, Spain and Sweden). Patient age and virus subtype/genetic clade information were available for 325 hospitalised patients from Finland, Greece, Ireland, the Netherlands, Norway, Portugal, Spain and Sweden (Figure 3B). Influenza subtypes and genetic clades associated with hospitalisation differed between age groups. Hospitalised cases in the 6–19 years age group and ≥ 65 years of age were most frequently infected by A/Texas/50/2012-like A(H3N2) viruses, 8/12 (67%; p = 0.0494) and 58/111 (52%; p = 0.0005), respectively (Figure 3B). All other hospitalised cases were infected in higher proportions by A/St Petersburg/27/2011-like (H1N1)pdm09 viruses, with rates of infection in children 6–19 (p = 0.0306) and adults ≥ 65 (p = 0.0011) years of age being significantly less than in 40–64 year olds.

##### Outcome

Among 521 of 1,633 cases with known disease outcome (alive/dead) from six countries (Greece, Ireland, Italy, Norway, Portugal and Spain), 41/521 (8%) died: 34/266 (13%) with A(H1N1)pdm09, 7/227 (3%) with A(H3N2) and 0/28 cases with type B influenza. Overall, A(H1N1)pdm09 infection occurred in 34 of 41 fatal cases. The majority of fatal cases were middle-aged and elderly adults: 20 were ≥ 65 years old and 12 were between 40 and 64 years old. One infant aged < 1 year (A(H1N1)pdm09 infected) and two children in the 6–19 age group (A(H3N2) infected) died. No further information was available for these patients.

##### Vaccination status

Vaccination status was known for 798 of the 1,633 cases from all 11 countries; 130 (16%) had been vaccinated with the influenza vaccine for the 2013/14 influenza season. Among these, there were 400 (50%) males and 396 (50%) females (two cases with unknown sex). Vaccination coverage ranged from 4% in children 1–5 years of age to 45% among those ≥ 65 years of age. None of the infants < 1 year of age had been vaccinated. Vaccination status and hospitalisation was known for 712 patients. Among 139 hospitalised cases, 34 (24%) had been vaccinated against influenza. Of those vaccinated and hospitalised, 20 had an A(H3N2), 12 an A(H1N1)pdm09 and two a B/Yamagata infection. Of the 16 fatal cases for which vaccination status was known, three had been known to be vaccinated. Two of these cases were infected by A(H3N2) and one by A(H1N1)pmd09 virus. Due to limited data completeness for outcome and vaccination status, no statistical analysis was performed.

##### Other epidemiological variables

Exposure to antiviral drugs was reported as known for 725 of 1,633 cases, and of these 576 (79%) had not been treated with antiviral drug. Of the cases reported, 492 of 521 (94%) were not immunocompromised and 29 had an underlying disease. The probable country of infection varied among the cases. For 669 (41%) cases this information was entered and 15 (2%) of the cases had probably acquired their infection during travel outside Europe (in Aruba, China, Indonesia, Israel and Saudi Arabia).

## Discussion

In this pilot study, TESSy was used to capture influenza virus strain-based antigenic and genetic characterisation data allowing phylogenetic analysis and reporting on the demographic information, outcome, vaccination status, immune status and the probable country of origin of the characterised viruses at the European level for the first time. Strain-based data analysis was feasible based on good data completeness for variables such as virus subtype, patient age and sex. Large and small countries from northern, southern and western parts of EU/EEA reported data and the target set for detailed characterisation of 10% of the viruses detected was achieved.

Although the distribution of (sub)types in our study was not exactly the same as the distribution in all EU/EEA countries [18], all (sub)types were covered both in our aggregate and strain-based data. We recognised from past years’ data that the proportions of different virus types/subtypes/lineages as well as the dominant type/subtype/lineage can vary between countries each season.

This pilot study showed that characterised viruses were congruent with guidance on targeted sampling for further characterisation: the data reported covered all age groups and had no sex bias. However, in our data, A(H3N2) viruses were slightly overrepresented among those selected for characterisation (16% vs 10% for all subtypes). A(H3N2) viruses have proved difficult to characterise antigenically in recent years [19] and therefore greater effort has been put into their characterisation.

In this pilot study, influenza virus types and subtypes did not affect the sexes differently, but did differ across age groups: A(H1N1)pdm09 viruses predominated in younger adults in the 20–64 years of age group as during the 2009 pandemic and in infants < 1 year, while A(H3N2) viruses predominated in patients aged ≥ 65 years, school-aged children and teenagers. Although vaccination status was reported, completeness was low for underlying diasease and immune status, and therefore no conclusions could be drawn on a possible effect of vaccination on the age distribution.

It will be of interest to follow the trend for age distribution among hospitalised cases over several seasons to better understand the age-distribution of influenza infection associated with severe infection by (sub)types and strains. For the 2012/13 season when type B viruses predominated across 12 European countries (partly overlapping with this study), children 5–14 years of age were mostly infected by B viruses while all other age groups showed an even distribution of influenza A and B viruses [7].

Phylogenetic analysis was performed to understand the evolution of the different sub(types) and lineages in comparison to the vaccine strains and over the season. Overall, in the 2013/14 season, the genetic variation of circulating viruses was limited and most of the viruses belonged to the same genetic category, and were closely related to each other, in their respective subtype/lineage. All A(H1N1)pdm09 viruses clustered in genetic subgroup 6B that contains viruses which we showed to remain antigenically similar to the vaccine virus A/California/7/2009 [20]. The A(H3N2) viruses have drifted through several influenza seasons and the study population confirmed that the viruses circulating in 2013/14 were closely related to the 2013/14 vaccine virus, A/Texas/50/2012, within genetic group 3C.1 but further evolution was seen by subdivision of viruses to 3C.2 and 3C.3 clusters. Interestingly, among the 2013/14 season H3 sequences studied here, there were no sequences already falling in the genetic 3C.2a subdivision which was associated with antigenic drift in the course of the 2014 southern hemisphere and 2014/15 northern hemisphere influenza seasons. Phylogenetic analysis of the B/Yamagata viruses confirmed likewise the clustering to two groups represented by the 2013/14 vaccine virus (B/Massachusetts/02/2012; clade 2) and the 2015/16 vaccine virus (B/Phuket/3073/2013; clade 3). The circulating B/Victoria viruses remained closely related to the B/Brisbane/60/2008 vaccine virus.

In this pilot study, 34/41 of fatal cases were related to A(H1N1)pdm09 infection, compared with 58% in eight countries reporting outcomes through hospital surveillance in 2013/14 [21]. In the hospital surveillance data, many of the influenza viruses are reported without subtype and therefore no exact comparison is possible. Overall, only 41 (3%) of the 1,633 viruses characterised were from fatal cases which does not show a bias of the data towards fatal case specimens being characterised. An earlier analysis of the 2013/14 season showed that fatal outcomes occured mostly in adults > 40 years of age [21]; this pilot study showed the highest number of deaths in those ≥ 65 years of age. Based on our limited data on severe infection, hospitalised cases affected by A(H3N2) virus infection were mostly school-aged children and the elderly, in line with the results of the meta-analysis for seasonal influenza [11].

Limitations of this study were that: only 11 of the 30 EU/EEA countries agreed to participate, and only three submitted data with indication of hospitalisation status with both non-hospitalised and hospitalised cases as most laboratories do not have the clinical information; and NICs aim for good representativeness of specimen selection but acknowledge selection biases and constraints in terms of: (i) characterisation of more A(H3N2) viruses as these viruses are currently drifting rapidly and have become more difficult to culture and characterise than A(H1N1)pdm09 viruses; (ii) capturing enough type B viruses to inform vaccine composition recommendations; (iii) increased interest in hospitalised and severe cases/deaths; (iv) limited resources and therefore focus on start, middle and end of season; (v) influenza surveillance systems may underestimate the cases in both ends of the age span due to healthcare seeking behaviour and sampling at outpatient clinics.

The extension of the existing antiviral strain-based reporting scheme with genetic and antigenic characterisation data was welcomed and supported by the pilot countries and it strengthens EISN as virological data reported can be subjected to more detailed analysis inclusive of the associated demographic and clinical information. We consider this as a substantial improvement over the previous aggregate reporting of antigenic and genetic categories only. Strain-based reporting also enabled early 2014/15 and 2015/16 influenza season analysis including HA phylogeny [22,23]. Through more traditional hospital surveillance, only virus subtype information related to hospitalisation has been reported by eight countries [24], but now genetic clade can be associated with information on hospitalisation.

We recommend the strain-based reporting to all EISN laboratories and we also recommend that laboratories continue to select specimens for characterisation across subtypes, geographic location and age groups, related to indicators of clinical status. The same principles as for selecting specimens to be sent to WHO CCs for detailed characterisation and informing vaccine composition recommendations may be adopted for national specimen selection [14]. Further, detailed reporting may allow greater definition of risk groups and support targeted vaccination and antiviral treatment strategies, e.g. if data on underlying conditions are included. The data should be combined with available hospital surveillance data as they may provide new ways of looking into vaccine effectiveness that has been low for A(H3N2) viruses in recent years [25].

The interplay between clinicians, epidemiologists and virologists collecting this type of data with public health specialists is crucial to ensure an even more representative sampling scheme for virus specimens. This will help to provide data for better estimates of risk factors associated with influenza.

## * Authors’ correction

The percentage marked with an asterisk was corrected, to 62%, at the request of the authors on 13 October 2016.
