# Authors’ correction for Euro Surveill. 2016;21(41)

**DOI:** 10.2807/1560-7917.ES.2016.21.42.30379

**Published:** 2016-10-20

**Authors:** Eurosurveillance editorial team

**Affiliations:** 1 European Centre for Disease Prevention and Control (ECDC), Stockholm, Sweden

In the article ‘Improving influenza virological surveillance in Europe: strain-based reporting of antigenic and genetic characterisation data, 11 European countries, influenza season 2013/14’, by Broberg et al., published on 13 October 2016, the percentage of influenza A(H1N1)pdm09 viruses in those aged  ≥ 65 years in the abstract was corrected (to 62%) post-publication, at the request of the authors on 13 October 2016.

